# CURE-AI: Contextualizing Uncertainty in Clinical Decision Making: A Conceptual Framework for Uncertainty-Aware AI Designs

**DOI:** 10.3390/healthcare14213703

**Published:** 2026-11-01

**Authors:** Dimitrios Zikos, Anuradha Chandrasekaran, Kewei Sha

**Affiliations:** 1 Department of Healthcare Management and Leadership, Texas Tech University Health Sciences Center, Lubbock, TX 49430, USA; 2 SHP Office of Research and Innovation, Texas Tech University Health Sciences Center, Lubbock, TX 49430, USA; anuradha.chandrasekaran@ttuhsc.edu; 3 Department of Data Science, University of North Texas, Denton, TX 76203, USA; kewei.sha@unt.edu

**Keywords:** clinical decision support, artificial intelligence, uncertainty quantification, explainable AI, socio-technical systems, medical decision making, workflow integration

## Abstract

**Highlights:**

**What are the main findings?**

- The CURE-AI framework formalizes the interaction between internal computational variance and external clinical ambiguity.
- Instead of simply reporting static confidence scores, AI uncertainty can be transformed into five practical clinical actions—such as identifying missing patient data, alerting clinicians to protocol conflicts, and recognizing natural biological unpredictability.

**What are the implications of the main findings?**

- Future clinical decision tools can act as collaborative partners, working back-and-forth with clinicians to show them what steps to take when a case is ambiguous.
- Mapping computational variance directly to clinical risk magnitude ensures AI systems defer to human judgment in high-stakes decision making scenarios.

**Abstract:**

Background/Objectives: Current clinical AI systems often report uncertainty as a static metric not tailored to the dynamic nature of clinical decision-making. To bridge the gap between computational variance and natural uncertainty in clinical practice, we propose the Contextual Uncertainty Reasoning for Artificial Intelligence (CURE-AI) framework. Methods: CURE-AI formalizes system uncertainty as a logical interaction between internal computational variance (the AI/ML pipeline) and external clinical ambiguity (the clinical workflow). The framework is constructed on five hierarchical levels of uncertainty resolution: (1) Clinical Uncertainty, (2) Computational Uncertainty, (3) Contextual Linkage, (4) Clinical Response, and (5) Human Outcome. Results: By mapping the framework to clinical workflows, we demonstrate that identical statistical uncertainty margins require different system recommendations depending on the patient context. CURE-AI translates computational variance into five actionable system behaviors: (1) inquiring for targeted missing data to resolve epistemic gaps, (2) accepting biological variability to prevent unnecessary testing, (3) scaling automated decisions or deferring to human judgment based on clinical risk, (4) suppressing AI output to adhere to mandated clinical protocols, and (5) recommending longitudinal patient reevaluation for prognostic uncertainty. Conclusions: CURE-AI recognizes AI uncertainty as an active workflow. By mapping computational variance to clinical action, the framework provides a conceptual blueprint for decision support systems that foster appropriate reliance and safeguard patient care.

## 1. Introduction

Current clinical decision support systems frequently rely on static metrics and representations of uncertainty, such as probabilities, confidence intervals, error bars, and heatmaps [1,2]. While these provide a baseline quantification, they represent a non-interactive communication of risk. These representations are limiting because they do not capture the dynamic nature of healthcare decision-making, where new patient information constantly enters the Electronic Health Record (EHR) and continuously changes the clinical picture [3]. While this is not an LLM-pipeline specific challenge, it becomes more prominent, when clinical decision support systems increasingly incorporate LLMs in their clinical decision support pipelines [4].

During patient care, clinicians make several decisions: ordering diagnostic exams, establishing diagnoses, selecting treatments, reevaluating clinical status, and determining readiness for discharge, transfer, or decide that the patient needs to be referred to a specialist of a different line of service. Each of these clinical decisions introduces their own uncertainty elements that impact patient safety and quality of care. For example, diagnostic ambiguity can lead to delayed interventions; aleatoric uncertainty (biological variability) during treatment can result in adverse events; and prognostic uncertainty during discharge evaluations can lead to unsafe readmissions or overuse of resources [5]. Because these sources of clinical uncertainty exist independently of AI and machine learning (ML), uncertainty-aware systems must be designed around how clinicians themselves naturally experience, process, and resolve uncertainty.

The objective of this paper is to provide to the research community (i) a comprehensive structured understanding of the mandate for contextually clinically relevant AI/ML uncertainty and (ii) a conceptual blueprint for future AI/ML clinical uncertainty-aware systems. In that respect, the manuscript sits at the intersection of clinical informatics and ML engineering. Our intended audience includes clinical informatics researchers and medical AI system designers. We establish a terminology used in the article: the computational AI/ML pipeline is referred to as the “internal system,” while the real-world clinical workflow it supports is referred to as the “external system.” It is important to emphasize that the goal of this work is not to propose specific algorithmic designs for uncertainty quantification. Our focus is to establish the abstraction layer of these designs, or in other words the required mechanisms to translate statistical variance from the internal system into contextually relevant and safe clinical decisions in the external system. Because CURE-AI is a conceptual framework and design blueprint, technical implementations discussed in Section 4.1 (e.g., deep ensembles) are provided as illustrative mechanisms for operationalizing this blueprint.

The structure of the article is as follows: The paper begins in Section 2 by decomposing the clinical decision-maker’s inherent clinical uncertainty and formalizing the intra- and inter-domain relationships between the external and internal systems. We then establish five specific linkage criteria that connect the internal and external systems to translate AI/ML pipeline variance into external clinical actions. Section 3 presents the resulting conceptual framework, which we named CURE-AI (Contextual Uncertainty REasoning for AI), applying these linkage criteria in a clinical scenario. Finally, Section 4 discusses the technical and architectural implementation considerations necessary to operationalize CURE-AI in clinical decision support designs.

To distinguish CURE-AI from prior work, Table 1 positions our proposed framework against models of clinical AI uncertainty. While existing paradigms, such as the epistemic/aleatoric taxonomy proposed by Begoli et al., the probabilistic bounding of Kompa et al., or the post-hoc explainability heatmaps by Ghassemi et al., treat uncertainty as a static metric. CURE-AI treats computational variance as a workflow change mechanism. It translates this variance into specific algorithmic interventions that interact with the clinical workflow, dictating subsequent actions and not just communicating risk to the clinical decision maker.

## 2. Materials and Methods

### 2.1. Decomposition of Clinical Decision-Maker Uncertainty

To develop a clinically relevant foundation for AI systems, we must first define the components of the human decision-maker’s uncertainty independent of AI and ML. In clinical practice, uncertainty can be decomposed into a horizontal space representing the continuum of care, intersected by two vertical dimensions that define the origin and meaning of that uncertainty which has also been discussed and introduced by others, such as Han et al. and Scott et al. [8,9]. The horizontal phases and vertical dimensions adapt established clinical taxonomies. CURE-AI’s contribution is mapping these dimensions to computational AI variance. It also attempts to specify the elements that need to be examined to understand the source (vertical dimension 1) and the interpretation (vertical dimension 2) of uncertainty. The horizontal space represents the phases of care where uncertainty occurs: diagnostic testing, establishing a diagnosis, treatment, and prognosis. Across this horizontal continuum, clinical uncertainty is driven and contextualized by two vertical dimensions:

Vertical Dimension 1: Source of Uncertainty. This dimension addresses why uncertainty exists in clinical practice. It is driven by three factors:

- The need for more data: Epistemic uncertainty that is typically controllable and reducible via additional diagnostic tests or patient history [10].
- Inherent probability of correct decision: Uncontrollable uncertainty reflecting humanity’s knowledge gaps, biological variability, and the probabilistic nature of medical science [6].
- Clinician-specific factors: Subjective uncertainty stemming from a lack of clinical experience.

Vertical Dimension 2: Interpretation of Uncertainty. This dimension addresses what a decision made despite uncertainty would mean in the real world. It encompasses:

- Criteria and Clinical Consequences: The evaluation of uncertainty based on the severity of the outcome and the potential harm the decision could trigger.
- Clinical Protocol Constraints: Uncertainty that is suppressed by mandated protocols of care, which dictate specific clinical pathways regardless of underlying ambiguity [11].
- Clinician Tolerance: This describes the individual, subjective clinician tolerance for uncertainty, which may stem from personality, experience, education and training, and prior professional experiences [12,13].

**Figure 1:**
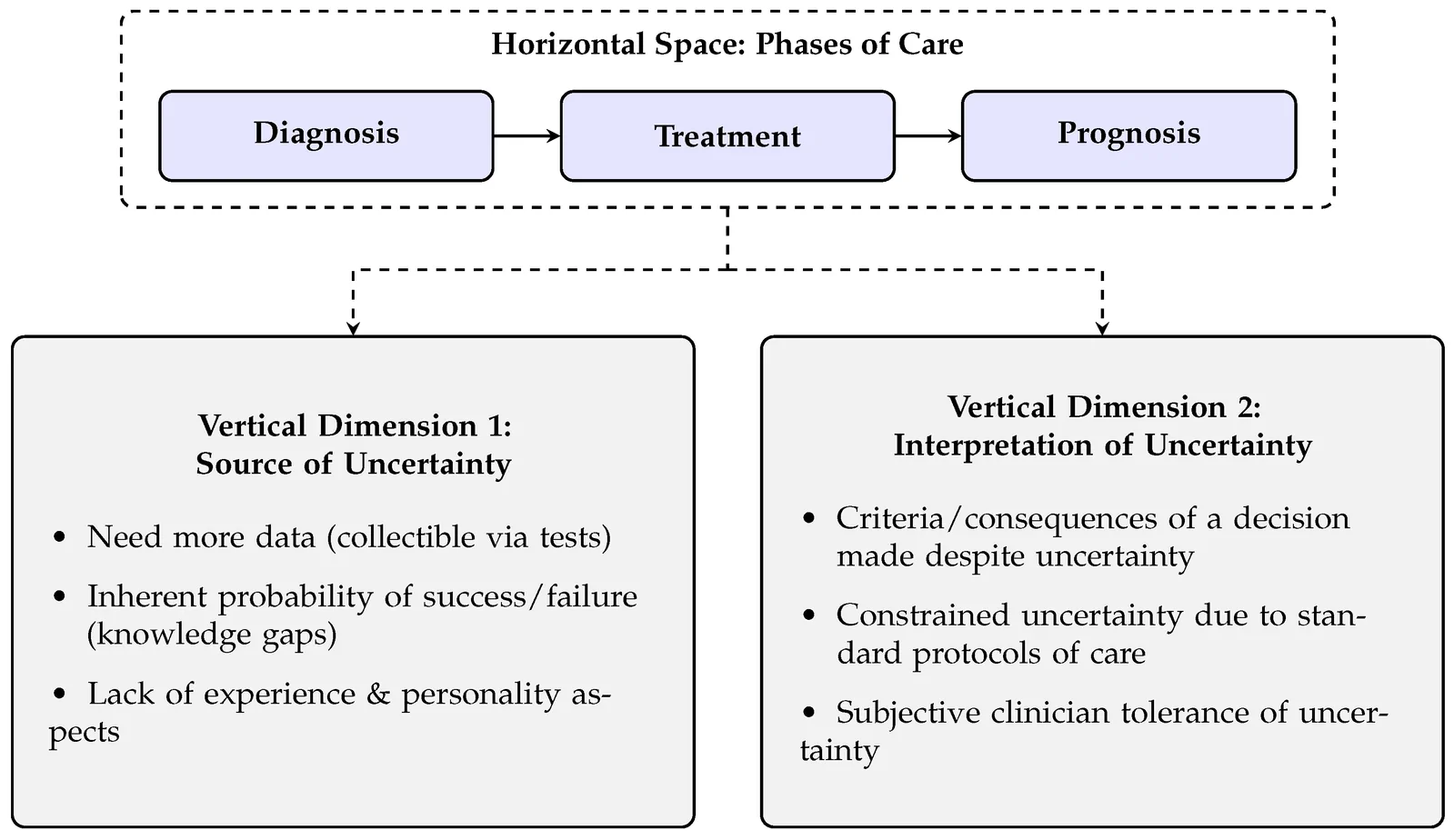
Decomposition of Clinical Decision-Maker Uncertainty, showing how the two vertical dimensions (Source and Interpretation) occur across the clinical care phases.

These dimensions establish that real-world clinical uncertainty is a structured process of identifying missing data, acknowledging knowledge limits, and weighing the consequences of action against oftentimes mandated clinical protocols [2,6]. Figure 1 illustrates how the source and interpretation of uncertainty happen across the clinical care phases.

### 2.2. Overall Design Approach of CURE-AI

The overarching design approach of CURE-AI relies on (a) connecting the real-world uncertainty experienced by the human decision-maker with the computational uncertainty of the AI system, and (b) connecting the components within the internal system (aka how uncertainty passes from the data-layer to the ML layer to the LLM layer), and connecting the components within the external system (aka, how uncertainty passes from the testing decisions to establishing a diagnosis to establishing the appropriate treatment). To achieve these connections, we will be examining uncertainty as a construct of two processes which we discuss in this paper as two systems: external (clinical) uncertainty and internal (AI/ML) uncertainty. And we will examine uncertainty within each of these two systems, and across the two systems (Table 1).

The rationale for separating and then connecting external clinical uncertainty and internal AI variance is supported by published work to bridge medical taxonomy with computational explainability. The decomposition of external clinical uncertainty into source, issue, and locus dimensions builds upon the taxonomy by Han et al. [8], who explain that clinical ambiguity is multi-dimensional [8]. Furthermore, Scott et al. explain that uncertainty in clinical practice cascades across the continuum from diagnosis to prognosis and treatment, validating the intra-domain clinical flow of CURE-AI [9]. Conversely, within the computational domain, reviews by López et al. and Salvi et al. identify the fragmentation of uncertainty quantification across isolated processing stages as a limitation in current ML pipelines [14,15]. The proposed approach to tracking internal intra-domain uncertainty (from data input through ML prediction to LLM generation) addresses this fragmentation because it treats the AI pipeline as a continuous inference chain. Finally, the inter-domain bridging of these two processes is supported by Fan’s work on integrating explainability with uncertainty estimation, where the authors explain that technical variance must be contextualized into clinically meaningful actions to support clinical trust and appropriate reliance [16].

### 2.3. Conceptual Formalization of Uncertainty

We define External Uncertainty as the AI/ML-independent clinical ambiguity in patient care. We conceptualize that this external domain operates across a horizontal space (the phases of care: testing, diagnosis, treatment, and prognosis) and is defined by two vertical dimensions: the source of the uncertainty (e.g., epistemic data gaps versus aleatoric biological variability) and the interpretation of that uncertainty (e.g., outcome severity and constraints due to mandatory clinical protocols of care). Conversely, we define Internal Uncertainty as the technical, computational variance from the AI-ML pipeline. This internal uncertainty can stem from data and measurement incompleteness, ML prediction variance, attribution and explanation instability, knowledge retrieval ambiguity, and LLM hallucination.

To formalize the uncertainty of an AI-assisted clinical decision system ($U_{Total}$), we can mathematize the relationships between the external (clinical) and internal (AI/ML) systems. The total system uncertainty must account for the requirement that the clinical workflow and the AI pipeline should be connected.

Therefore, $U_{Total}$ is conceptualized as the aggregate of the intra-domain uncertainties, modified by an inter-domain interaction term:

(1) $$U_{Total}=U_{Intra}^{External}+U_{Intra}^{Internal}+U_{Inter}$$

1. Intra-Domain External Uncertainty ($U_{Intra}^{External}$): This represents the uncertainty that exists in the clinical process. It combines the source of clinical uncertainty (*S*) and its interpretation or clinical consequence (*C*) across the horizontal phases of clinical decision process (*p*):

(2) $$U_{Intra}^{External}=\sum_{p=1}^{n}f\left(S_{p},C_{p}\right)$$

While Equation (2) is presented as a summation across phases of care (*p*), this represents the *accumulation* of unresolved uncertainty. If diagnostic uncertainty is not resolved early, it acts as a baseline that inflates the complexity of treatment selection, representing the ‘domino’ effect discussed in Section 2.6.1.

2. Intra-Domain Internal Uncertainty ($U_{Intra}^{Internal}$): This represents the algorithmic variance through the AI/ML pipeline. Reflecting the dependent inference chain from data input, to ML prediction, to LLM generation, it aggregates the uncertainty across these layers of the AI/ML pipeline ($L_{i}$):

(3) $$U_{Intra}^{Internal}=\sum_{i=1}^{k}\sigma^{2}\left(L_{i}|L_{i-1}\right)$$

Equation (3) conceptualizes the internal pipeline as a Markovian chain. By utilizing conditional variance $\sigma^{2}\left(L_{i}|L_{i-1}\right)$, the formula accounts for amplification and attenuation: variance at the LLM layer is dependent on the stability of the preceding Knowledge Retrieval and ML layers. An unstable upstream layer amplifies downstream uncertainty.

3. Inter-Domain Uncertainty ($U_{Inter}$): This is the interaction modifier between the internal (the AI/ML pipeline) and the external system (the clinical process). Because internal AI variance and external clinical ambiguity are not independent, they cannot be simply added together without accounting for their interaction. $U_{Inter}$ captures the degree of alignment between the two systems, and can be represented similar to covariance interaction:

(4) $$U_{Inter}=2\cdot \mathrm{Cov}\left(U_{Intra}^{Internal},U_{Intra}^{External}\right)$$

The covariance term in Equation (4) is a high level representation and not meant to be a computable parameter. It represents why computational variance cannot be isolated from clinical context. If AI uncertainty aligns with clinical context (e.g., both recognize a test is missing), covariance allows the system to trigger a safe resolution pathway. If they are misaligned, the interaction term increases overall uncertainty.

If the CURE-AI framework successfully applies these criteria (such as translating an internal data gap into an clinical request for a missing lab test) the internal and external systems are likely to be better aligned. In this scenario, $U_{Inter}$ acts as a negative covariance, reducing $U_{Total}$ and reducing overall ambiguity. On the other hand, if the AI output is misaligned with mandated clinical protocols or presents unresolvable ambiguity, $U_{Inter}$ acts as a penalty, inflating the total uncertainty beyond the sum of its parts.

These formulas serve as a formal conceptual representation of the dependencies that a clinical decision-support implementation must take into account. They are not quantitative estimators of a specific uncertainty metrics. The CURE-AI’s primary contribution is in defining the linkage logic between computational variance and clinical workflow. This is why we are formalizing these relationships: to establish the blueprint for how AI/ML uncertainty interacts with human cognition.

### 2.4. Defining the Uncertainty Linkage Criteria

**Figure 2:**
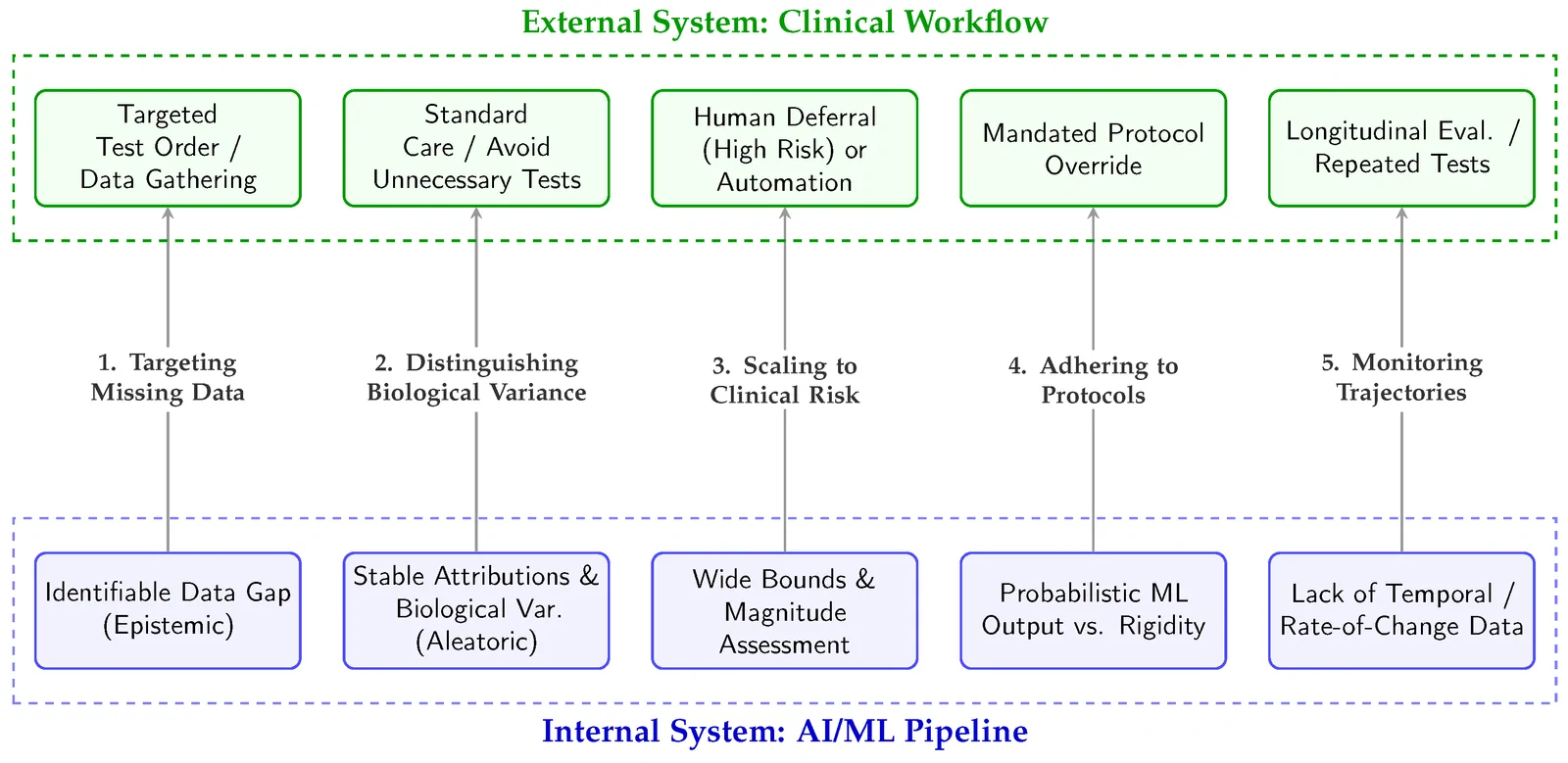
Abstracted Internal–External System Interaction. The figure shows how specific internal computational uncertainty states (**bottom**) are translated across the five inter-domain linkage criteria into external clinical actions (**top**).

Now that we have formalized this interaction, the challenge for CURE-AI is establishing the practical criteria to link that internal system uncertainty to the external system (clinical) uncertainty. We hereby define rules of interaction between the AI/ML and the external clinical workflow (Figure 2).

We propose five inter-domain linkage criteria that focus on clinician behaviors: (1) Targeting Missing Data, (2) Distinguishing Biological Variance from Resolvable Uncertainty, (3) Scaling to Clinical Risk, (4) Awareness of Clinical Protocols, and (5) Monitoring Physiological Value Trends. These criteria determine how specific categories of computational uncertainty correspond/translate into clinical actions (Table 2). We will utilize these criteria to define specific user-system interactions in the Results section. To apply these linkage criteria, however, we need to isolate where and how uncertainty originates in the internal system (AI/ML pipeline), Table 3.

### 2.5. Formalizing Inter-Domain Linkage Logic

To operationalize the connection between internal AI/ML uncertainty and external clinical action, we define linkage criteria as logical rules. Let $U_{epi}$ represent epistemic uncertainty, $U_{ale}$ represent aleatoric uncertainty, $D_{missing}$ denote the set of missing input variables, $A_{stable}$ represent the stability of feature attributions, $R_{clinical}$ denote the risk magnitude of the clinical decision, and $C_{protocol}$ represent a mandatory clinical protocol.

Thresholds such as $\tau_{epi}$, $\tau_{safety}$, and $\tau_{bypass}$ are not meant to be global constants but instead they must be calibrated by each hospital/unit based on departmental acuity (e.g., ICU vs. outpatient) and validated via hospital specific EHR data that represent the unique case mix of each organization. For example, parameters such as $R_{clinical}$ could be represented by standard acuity scoring systems (e.g., APACHE II or MEWS) or any other system deemed appropriate, integrated into the threshold logic.

#### 2.5.1. Targeting Missing Data (Linkage Criterion 1)

When epistemic uncertainty exceeds a threshold ($\tau_{epi}$) and the algorithm identifies missing inputs, the system triggers a request for additional data.

(5) $$\mathrm{IF}\;\left(U_{epi}>\tau_{epi}\right)\wedge \left(D_{missing}\neq \emptyset \right)\;\mathrm{THEN}\;\mathrm{Action}=\mathrm{Acquire}\left(D_{missing}\right)$$

#### 2.5.2. Distinguishing Biological Variance (Linkage Criterion 2)

If aleatoric uncertainty exceeds its threshold ($\tau_{ale}$), but the input data is complete and the underlying feature attributions are stable, the uncertainty is irreducible variance. The system suppresses further diagnostic testing informing the clinician that the uncertainty is uncontrollable.

(6) $$\mathrm{IF}\;\left(U_{ale}>\tau_{ale}\right)\wedge \left(D_{missing}=\emptyset \right)\wedge \left(A_{stable}=\mathrm{True}\right)\;\mathrm{THEN}\;\mathrm{Action}=\mathrm{AcceptVariance}$$

#### 2.5.3. Scaling to Clinical Risk (Linkage Criterion 3)

The total uncertainty of the pipeline ($U_{total}$) must be weighted by the clinical consequence of the decision ($R_{clinical}$) [6]. Furthermore, to address the vertical dimension of Clinician Tolerance (Figure 1), the maximum allowable safety threshold ($\tau_{safety}$) should not be a fixed constant. In a clinical interface, $\tau_{safety}$ can be implemented as a role-based parameter. For example, an attending specialist may operate with a wider $\tau_{safety}$ bound for automated triage than a medical resident with less experience. When the risk-weighted uncertainty exceeds this profile-specific safety threshold, the system defers to human judgment.

(7) $$IF(U_{total}\times R_{clinical}>\tau_{safety})\;\mathrm{THEN}\;\mathrm{Action}\;=\;\mathrm{DeferToHuman}$$

#### 2.5.4. Criterion 4: Awareness of Clinical Protocols

Clinical protocols act as evidence based practice instructions, but they can sometimes conflict with a patient’s unique circumstances. The AI system is not designed to override these hospital rules. Instead, if the AI calculates that following the standard protocol might actually put the patient at risk, it flags the mismatch. This prompts the clinician to step in and decide whether to deviate from the standard protocol. This keeps the human in control; the AI acts as a safety net, while any departure from protocol remains clinician-led.

(8) $$\begin{array}{cc} \; & \mathrm{IF}\;\left(C_{protocol}==Active\right)\wedge \left(P_{AI}\neq C_{protocol}\right)\;\mathrm{THEN}:\\ \; & \;\mathrm{IF}\;(Risk\left(P_{AI}\right)>\tau_{bypass})\;\mathrm{THEN}\;\mathrm{Action}\;=\;\mathrm{EscalateConflict}(\mathrm{Clinical}\_\mathrm{Supervisor})\\ \; & \;\mathrm{ELSE}\;\mathrm{Action}\;=\;\mathrm{Suppress}(P_{AI}) \end{array}$$

When escalated, the AI system stops automated decision-making and requires human override by the clinical decision maker to bypass the protocol.

#### 2.5.5. Monitoring Physiological Value Trends (Linkage Criterion 5)

Let $U_{prog}$ represent prognostic uncertainty and $\Delta t(Data_{obs})$ be the time span of available longitudinal data. If prognostic uncertainty is high and the observation window is shorter than the required clinical trending period ($T_{trend}$), the system triggers a request for additional data points to create reliable trend-lines.

(9) $$\mathrm{IF}\;\left(U_{prog}>\tau_{prog}\right)\wedge \left(\Delta t\left(Data_{obs}\right)<T_{trend}\right)\;\mathrm{THEN}\;\mathrm{Action}=\mathrm{DelayAndReevaluate}$$

### 2.6. Intra-Domain Dependencies

#### 2.6.1. Dependencies Within External (Clinical) Uncertainty

In the clinical domain, uncertainty exists across the continuum of care and interacts with the vertical dimensions. Diagnostic ambiguity compounds treatment uncertainty; if a clinician is unsure of the exact etiology (Horizontal Phase 1), selecting the optimal targeted therapy (Horizontal Phase 2) carries a higher risk.

Furthermore, the vertical dimensions are intertwined. The source of uncertainty dictates its interpretation and the resulting clinical action. For example, if the source of uncertainty is a lack of clinician experience or subjective hesitation (Vertical Dimension 1), an clinical protocol (Vertical Dimension 2) acts as a constraint, and overrides uncertainty, and it turn forces a standardized decision. External uncertainty is therefore like a “domino” of clinical logic.

#### 2.6.2. Dependencies Within Internal (AI/ML) Uncertainty

Similarly, internal AI uncertainty propagates sequentially through the AI/ML pipeline. The AI pipeline functions as a continuous sequence: Data → ML Model → Attribution → Knowledge Retrieval → LLM Narrative. Uncertainty can be amplified, attenuated, or transformed across these pipeline layers.

A failure or data gap in an early step invariably passes uncertainty to the later steps. For instance, missing inputs at the data layer manifest as prediction variance at the ML layer. This predictive instability then cascades into the attribution layer, producing fluctuating feature-importance scores. When this unstable attribution is fed into a generative LLM interface, it increases the risk of hallucination in the clinical narratives [7].

### 2.7. Synthesis Methodology

With the clinical and computational domains formalized, their dependencies mapped, and their inter-domain linkage criteria established, we now synthesize these elements into a conceptual framework for uncertainty-aware AI systems. Our synthesis methodology maps the sequential stages of the internal AI pipeline (Data→ML→Attribution→ Knowledge→LLM) to the dimensions of the external clinical workflow. This is essentially the representation of CURE-AI. The framework was developed through heuristic mapping: we isolated known computational failure states (e.g., data sparsity, output instability) and mapped them to typical clinical mitigation strategies (e.g., ordering tests, “wait and see” approaches) widely applicable in clinical practice.

## 3. Illustrative Application

### 3.1. The Conceptual Framework for Contextual Uncertainty Reasoning

**Figure 3:**
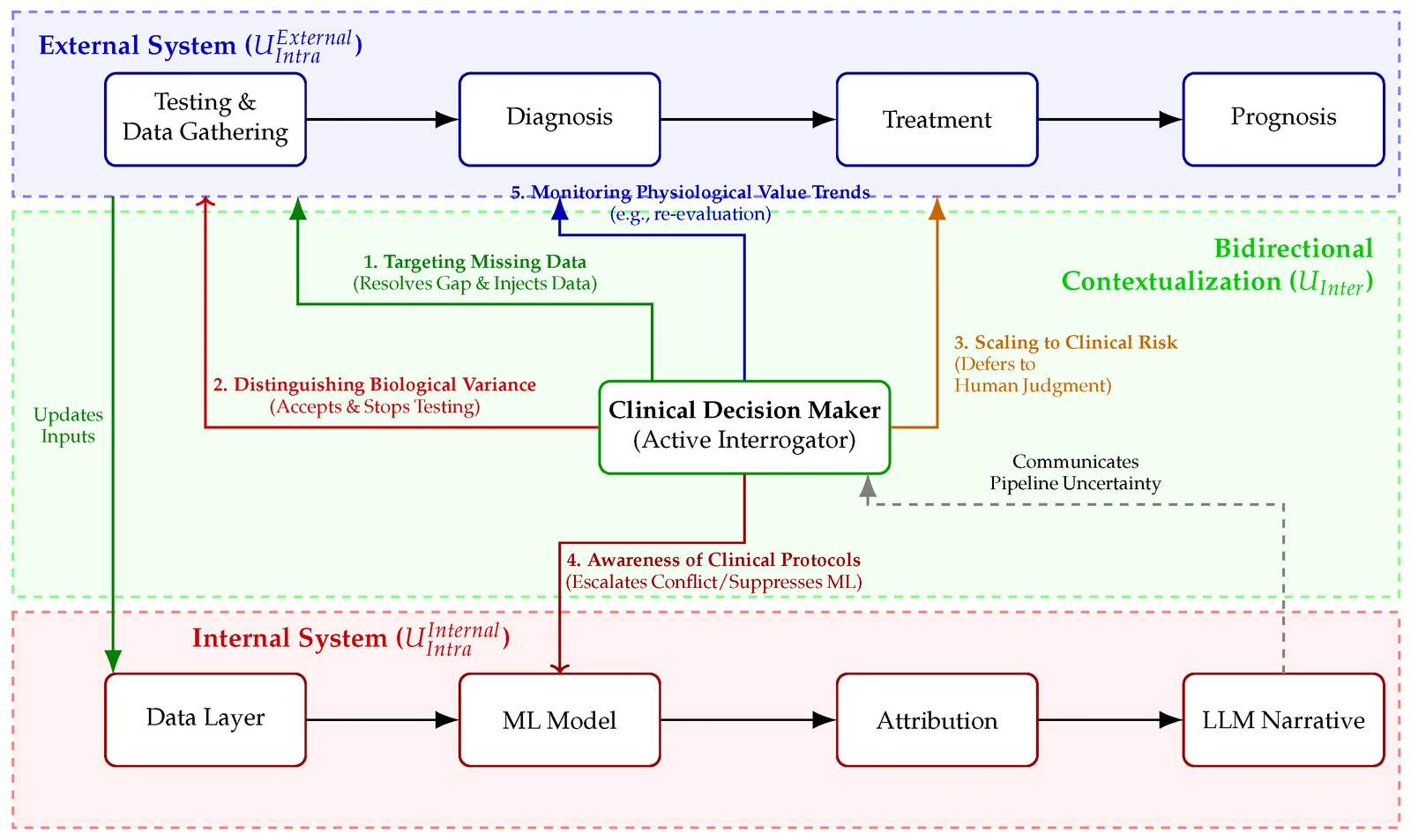
The Conceptual Framework for Contextual Uncertainty Reasoning, illustrating the bidirectional loop ($U_{Inter}$) that bridges the internal AI pipeline with the external clinical workflow.

The resulting CURE-AI represents the conglomeration of these contextual characteristics, linking AI pipeline uncertainty to clinical uncertainty processes (Figure 3). The clinical decision-maker becomes an active interrogator of the AI/ML narrative outputs. CURE-AI facilitates a bidirectional loop between the internal AI/ML system and the external clinical workflow during clinical care, through five interaction mechanisms. With the mapping of internal algorithmic uncertainty to clinical logic, the system translates uncertainty into clinical actions: (1) acquiring missing data, (2) accepting biological variance, (3) deferring decisions according to clinical risk, (4) being cognizant of mandated clinical protocols, and (5) monitoring physiological value trends.

### 3.2. Applying the Linkage Criteria via a Clinical Scenario

Based on the inter-domain linkage criteria established in our methodology, we establish the following linkage examples via a clinical scenario. This scenario shows how internal AI/ML uncertainty is translated into specific clinical actions. This scenario is a hypothetical construct developed to sequentially illustrate the framework’s mechanics. We acknowledge that its clinical plausibility has not yet been validated with prospective clinical evaluation.

#### 3.2.1. Collaborative Clinical Reasoning and Bidirectional Interrogation

For an AI system to be clinically useful, its uncertainty representation should follow the natural cognitive processes of the clinical decision-maker. This requires a collaborative, bidirectional human–computer interaction where clinicians become reasoners who can interrogate the system. A clinically integrated AI must allow the user to ask why the system is uncertain, which specific pipeline component is responsible, and what and whether new evidence would reduce that uncertainty. With that consideration in place, clinicians can test counterfactual queries and supply new data (e.g., by additional lat tests), prompting the system to recalculate and update its uncertainty representation. To prevent unwarranted reliance during this interaction, any clinical recommendation or uncertainty explanation communicated within an LLM narrative must be traceable to established domain knowledge, underlying ML measures, data completeness, or mandated clinical protocols. To illustrate this bidirectional interrogation, consider the difference in narrative outputs:

Standard LLM Output: “Patient presents with hyperglycemia. Initiate long-acting insulin therapy.”

CURE-AI LLM Output: “Hyperglycemia is probable. However, due to high epistemic uncertainty ($\tau_{epi}$) due to missing 12-h CGM synchronization, treatment recommendation is paused. Please sync CGM data to resolve this variance before dosing.”

#### 3.2.2. Targeting Missing Data via Real-World Clinical Queries

During the initial stages of care, clinicians frequently face epistemic (controllable and reducible) uncertainty due to missing data. Consider a 65-year-old patient with chronic type 2 diabetes and diabetic nephropathy admitted to the hospital with mild confusion. The AI system evaluates the patient for hyperglycemia risk. It calculates a high probability but flags the prediction with high epistemic uncertainty. Instead of outputting a generic warning or providing a low-confidence prediction, the system abstains. It specifies that the uncertainty is driven by insufficient data: the continuous glucose monitor (CGM) has not been synced and the metabolic panel is missing. The system therefore prompts the clinician to order a STAT metabolic panel and sync the CGM.

#### 3.2.3. Navigating Clinical Protocols

Treatment selection is frequently driven by hospital protocols, which act as rules that override AI uncertainty. Once the new labs arrive, they indicate acute renal decline, specifically a low estimated Glomerular Filtration Rate (eGFR). The AI initially calculates a standard long-acting insulin dose for the patient’s blood sugar. However, it cross-references the patient’s updated lab profile against the hospital’s renal protocol. Recognizing that mandatory guidelines take priority, the system flags the conflict before any automated dosing occurs. It warns the clinician that the standard calculated dose is against the renal protocol and proposes a protocol-compliant adjusted dose.

#### 3.2.4. Distinguishing Biological Variance

When evaluating patients, an uncertainty-aware system must recognize when it lacks the reliability to make a precise prediction due to uncontrollable biological variability (aleatoric uncertainty). After the data profile is complete and the protocol conflict is flagged, the system attempts to predict the patient’s exact glycemic response to the adjusted insulin dose. The AI shows high variance in this prediction. CURE-AI identifies this as inherent biological variability, which is not modifiable and not resolvabile with more data. It prevents the generation of further unnecessary diagnostic test recommendations and prompts the clinician to accept the variance as a biological baseline, reflecting the clinician’s capacity to safely state “I don’t know.”

#### 3.2.5. Contextualizing Uncertainty in Terms of Clinical Consequences

Finally, the consequences of uncertainty must be evaluated against the potential severity of the clinical outcome. The magnitude of uncertainty should be weighed against the potential harm, prioritizing human review based on actual clinical risk. In our scenario, standardizing insulin titration in the presence of acute renal decline carries a high risk of hypoglycemia. Because this clinical consequence ($R_{clinical}$) is severe, the threshold for automated action is strict. Although the epistemic gaps were resolved, the system calculates that the total risk still exceeds the safety threshold ($\tau_{safety}$) for automated dosing. It presents the evidence and *defers* the final dosing decision to the clinician’s judgment.

#### 3.2.6. Monitoring Physiological Value Trends (Longitudinal Evaluation)

Finally, uncertainty in prognosis or treatment efficacy cannot be resolved with a single blood glucose cross-sectional data point. If prognostic uncertainty remains high due to a lack of temporal trend data, the decision pathway triggers a requirement for longitudinal observation. For our diabetes patient, the system flags that establishing the efficacy of the new insulin dose and type requires chart data for the blood sugar fluctuation. This, in turn, triggers a prompt for the clinician to schedule a follow-up to evaluate the CGM trend which in turn will feed the internal system with longitudinal data; this will reduce its internal uncertainty as a result.

### 3.3. Calibrated Trust and Reliance

Ultimately, CURE-AI’s clinical utility is achieved through observable clinician behaviors: the decision to accept, verify, seek evidence, override, or defer. The desired outcome of communicating pipeline-wide uncertainty is to strengthen reliance and calibrated trust [17]. This is why technical uncertainty needs to be bridged with clinical reasoning; to empower the clinician to understand when an AI output is a reliable extension of their own cognition, and when human judgment must intervene.

### 3.4. Synthesis of Context-Driven System Behaviors

While the five criteria outline the theoretical pathways of the framework, its operational value lies in translating AI uncertainty into a definitive set of systemic outputs. Ultimately, CURE-AI presents the clinical decision support system as an active workflow engine. Table 4 synthesizes how specific states of internal AI/ML uncertainty, when contextualized by the external clinical environment, generate useful and actionable system behaviors.

## 4. Discussion

The most significant conceptual implication of CURE-AI is that computational uncertainty generated by an AI model must lead to different system behaviors depending on the clinical context. The clinical phase, data availability, underlying biological variability, protocol rigidity, and the patient’s physiological value trends dictate the meaning of the AI’s variance.

Consider a hypothetical AI clinical recommender system that outputs a 20% uncertainty bound. Under CURE-AI, this static number is meaningless until it is contextualized through the external clinical situation (the external system as we call it):

- Context of Clinical Risk: A 20% uncertainty bound in a low-consequence triage decision may be deemed acceptable, allowing the system to proceed with an automated recommendation. However, that identical 20% uncertainty in a high-consequence intervention (e.g., surgical oncology) triggers deferral to human judgment (Criterion 3).
- Context of the Uncertainty Source: A 20% uncertainty driven by a single, easy to collect missing lab value prompts the system to generate an actionable data request (Criterion 1). Conversely, the exact same 20% uncertainty if it exists due to aleatoric biological variability prompts the system to advise the clinician to manage the patient via standard care without additional and likely unneeded lab tests and other examinations (Criterion 2).
- Context of the Clinical Phase: A 20% uncertainty during an initial diagnostic workup justifies exploratory testing. Yet, the same 20% uncertainty during a discharge or prognostic phase may simply trigger a recommendation for longitudinal outpatient monitoring (Criterion 5).

With these examples, we wanted to show that CURE-AI establishes that 20% computational uncertainty is not a monolith but it facilitates and produces the precise response required by the human decision-maker.

### 4.1. Technical and Implementation Considerations

Translating the inter-domain linkage criteria into a functional clinical AI pipeline requires specific technical architecture decisions. The bidirectional loop and continuous contextualization require a shift from traditional linear, static ML models to dynamic, interactive inference engines [18].

First, realizing the bidirectional interrogation loop necessitates real-time, low-latency uncertainty quantification (UQ). Current UQ methods, such as deep ensembles [19], perturbation analyses, or Monte Carlo dropout [20], are computationally heavy [21]. Because CURE-AI requires recalculating uncertainty dynamically as the clinician inputs new data or tests, the underlying architecture must utilize computationally efficient UQ estimators to prevent latency from disrupting the fast paced clinical workflow. Acceptable latency bounds vary by phase: for example <2 s for ED diagnostic triage to prevent workflow disruption, versus <30 s for retrospective prognostic analysis.

Second, a contextually aware AI/ML pipeline must be engineered to support cross-layer dependency tracking. Standard AI pipelines typically pass only the final predictive point estimate from the ML model to the generative interface. To achieve pipeline-wide uncertainty awareness, the system must pass uncertainty metadata, including data missingness flags, feature attribution stability scores, and prediction intervals, across all interfaces. This ensures that the downstream LLM narrative is grounded in the exact variance generated by the upstream data and ML, rather than treating each component as an isolated black box. Passing this metadata requires adopting interoperability standards, such as utilizing HL7 FHIR ‘RiskAssessment’ or ‘Observation’ extensions to carry model variance alongside the clinical data. Explaining specific interoperability considerations is beyond the scope of this article.

Third, operationalizing the mathematical linkage logic requires a deterministic rules engine (e.g., ontological networks) between the probabilistic ML models and the generative AI interface. This rules engine must continually monitor the specified uncertainty thresholds ($\tau_{epi}$, $\tau_{ale}$, $\tau_{safety}$) against the clinical risk magnitude ($R_{clinical}$) and clinical protocols ($C_{protocol}$). By hardcoding these linkage criteria as deterministic logic gates, the system can enforce safety overrides, suppress probabilistic outputs in favor of mandated care pathways, and trigger human deferrals without relying on the LLM’s non-deterministic reasoning. Because hardcoded clinical logic (Criterion 4) are sensitive to changing guidelines, accountability for maintaining the rules engine must fall to the hospital’s governance board.

Finally, the system’s text generation must be controlled to ensure traceability. Because LLMs naturally produce fluent and confident text, they can easily mask underlying uncertainty and inflate their perceived competence [7]. To counter this, the AI’s output should rely on guardrails and predefined templates [22] directly linked to the system’s uncertainty data. This prevents the model from generating false certainty, ensuring the final text reflects the AI’s actual confidence level.

### 4.2. Limitations and Future Validation

Because this manuscript presents a foundational, theoretical framework, its primary limitation lies in its intentionally conceptual scope. CURE-AI is designed as an architectural blueprint for future clinical decision support systems, mapping the theoretical translation of computational variance into clinical workflow. Consequently, this study does not include empirical validation, direct stakeholder appraisal, or quantitative measures of calibrated trust (e.g., reliance versus compliance rates). Furthermore, because the framework’s logic is illustrated via a single scenario, its generalizability across diverse clinical specialties (e.g., critical care versus outpatient psychiatry) is theoretically and not empirically grounded.

For CURE-AI to be an established applied clinical tool, future research must focus on empirical evaluation. A minimal validation of CURE-AI requires a retrospective EHR simulation to test our formalizations, followed by a usability study with clinicians. Our theoretical claims are scientifically falsifiable: if clinicians using CURE-AI interfaces do not demonstrate a statistically significant reduction in inappropriate testing (Criterion 2) or protocol violations (Criterion 4) compared to standard AI systems, the core premise of our mapping would require revision.

## 5. Conclusions

Clinical AI should not just quantify how uncertain its prediction is; it must determine what that uncertainty means in the current clinical context and translate it into an appropriate clinical action. This paper presented a conceptual framework that introduces an abstraction layer to achieve this goal. We established linkage criteria between the computational variance of the internal system and the real-world workflow of the external system, and we provide a blueprint for AI designs that align with natural clinical reasoning. To conclude, CURE-AI can be synthesized into a five-level hierarchy that defines the operational mandate for future uncertainty-aware systems:

1. **Clinical Uncertainty (Level 1):** Identifying what inherent ambiguity exists in the patient’s care independent of the AI.
2. **Computational Uncertainty (Level 2):** Tracking where technical variance arises inside the sequential AI pipeline.
3. **Contextual Linkage (Level 3):** Determining what the computational uncertainty means within the specific clinical situation and protocol constraints.
4. **Clinical Response (Level 4):** Facilitating the appropriate action (e.g., acquire missing data, accept biological variance, follow mandated protocols, reevaluate longitudinally, or escalate to human judgment).
5. **Human Outcome (Level 5):** Empowering the clinician to make an appropriately informed decision.

Future uncertainty-aware AI systems must prioritize this contextual translation to ensure that AI uncertainty metrics empower clinicians to resolve data gaps, reduce or understand ambiguity and ultimately make better clinical decisions.

## Figures and Tables

**Table 1:** Comparison of Clinical AI Uncertainty Frameworks.

Framework	Primary Representation of Uncertainty	Translation to Clinical Action	Integration with Clinical Workflow
Kompa et al. (2021) [6]	Probabilistic bounding and confidence intervals.	Non-actionable; relies on manual clinician interpretation.	Passive (communicates risk at point of prediction).
Begoli et al. (2019) [1]	Taxonomy of epistemic vs. aleatoric uncertainty.	Conceptual mapping of error sources without workflow triggers.	None (theoretical categorization).
Ghassemi et al. (2021) [7]	Explainability heatmaps and feature attribution.	Recognizes contributing data, but does not inform actions in turn.	Post-hoc analysis (evaluating a completed prediction).
CURE-AI (Proposed)	Bidirectional mapping of computational variance to clinical context.	Translates variance into algorithmic behaviors (e.g., test suppression, protocol escalation).	Active (intervenes during data gathering, treatment, and prognosis).

**Table 2:** Conceptual Matrix of Uncertainty Connections within and across Clinical and AI Domains.

Interaction Level	Process Domain	Description
Intra-Domain (Within the External System)	External (Clinical) Uncertainty	Tracks how uncertainty propagates across the clinical workflow, passing from initial testing decisions, to establishing a diagnosis, to establishing the appropriate treatment.
Intra-Domain (Within the Internal System)	Internal (AI/ML) Uncertainty	Maps how computational uncertainty moves through the AI pipeline layers, from the data layer, to the ML layer, and to the LLM layer.
Inter-Domain (Across Systems)	Connecting External and Internal	Connects the real-world uncertainty experienced by the human decision-maker with the computational uncertainty generated by the AI system, translating pipeline variance into clinically actionable metrics.

**Table 3:** Inter-Domain Linkage Criteria to Bridge Internal AI Uncertainty with Clinical Action.

Linkage Criterion	Internal AI Pipeline Definition	External Clinical Implication
1. Targeting Missing Data	High epistemic uncertainty driven by one or more identifiable missing variables at the data layer.	Generates a request for the clinician to order a specific test or gather information.
2. Distinguishing Biological Variance	High aleatoric uncertainty driven by biological variability: the data layer is complete and attributions are stable.	Prompts the clinician to accept the variance and manage the patient via standard care.
3. Scaling to Clinical Risk	The magnitude of AI uncertainty is weighed against the potential harm of the proposed clinical intervention.	High-risk decisions with wide uncertainty bounds trigger human deferral; low-risk decisions may be automated.
4. Awareness of Clinical Protocols	Probabilistic ML outputs conflict with or attempt to bypass mandated clinical pathways.	Mandated protocols of care override uncertain AI recommendations to ensure baseline patient safety.
5. Monitoring Physiological Value Trends	Prognostic uncertainty caused by a lack of rate-of-change or longitudinal data.	Triggers re-evaluations/repeated tests to measure trend for disease progression or treatment effectiveness.

**Table 4:** Matrix of Contextual Linkage and Generated System Behaviors.

Internal AI State	External Clinical Context	Generated System Behavior
Missing or sparse data inputs	Epistemic (Recoverable data gap)	Acquire: Generate targeted request for specific labs/imaging.
Complete data with high variance	Aleatoric (Biological variability)	Accept: Suppress further testing; manage via standard care.
High prediction uncertainty	High clinical consequence (Risk-scaled)	Defer: Halt automated pathways; escalate to human judgment.
Model output diverges from standard	Rigid protocol governs pathway	Escalate: Surface protocol–patient mismatch for clinician review.
Insufficient temporal data	Prognostic or discharge phase	Reevaluate: Mandate longitudinal outpatient monitoring.

## Data Availability

No new data were created or analyzed in this study. Data sharing is not applicable to this article.
